# Supplementary material for: Knowledge and determinants of infection prevention and control compliance among nurses in Yendi municipality, Ghana
Source: PLoS One. 2022 Jul 20;17(7):e0270508. doi: 10.1371/journal.pone.0270508 (PMC9299325; doi:10.1371/journal.pone.0270508)
Supplement: S1 Questionnaire — (PDF) [file pone.0270508.s001.pdf]

COLLEGE OF HEALTH SCIENCES, YENDI

DEPARTMENT OF REGISTERED GENERAL NURSING

Dear Respondent,

We are students from the College of Health Sciences, Yendi. The aim of this study is to assess '**Knowledge and determinants of infection prevention and control compliance among nurses in Yendi municipality, Ghana'**. The data obtained is to assist in completing our project work as a partial fulfillment for the diploma program. Your participation is voluntary to answer the following questionnaire. Your answer will be confidential. The result will be used to improve method of education and communication to nurses about their health regarding Infection Prevention and Control. The information will be treated with utmost confidentiality. Please answer the following questions and choose one answer that best expresses your opinion by **circling** the relevant number/fill in.

Do I have your consent? Yes [ ] No [ ]

QUESTIONNAIRE

SECTION A – Socio-demographic Characteristics

S/No	Question	Options
**D1**	What is your age in years?	……………………………………….
**D2**	What is your gender?	Male……………………………………........1<br>Female………………………………….…...2
**D3**	What is your professional rank?	…………………………………………
**D4**	Which department do you work?	…………………………………………
**D5**	What is your highest education level?	Certificate…………………………...……1<br>Diploma……………………..…………2<br>Degree…..…………………..……………3
**D6**	What is your years of practice?	…………………………………………….
**D7**	Have you had IPC training before?	Yes………………..………………………1<br>No ……………………….……………......2
**D8**	Do you have IPC committee in your facility?	Yes………………..………………………1<br>No ……………………….……………......2
**D9**	Do you have PPE's available in your facility?	Yes………………..………………………1<br>No ……………………….……………......2
**D10**	Do you have IPC guideline in your working department?	Yes………………..………………………1<br>No ……………………….……………......2

SECTION B – Knowledge on Infection Prevention and Control

S/N	Question	Options
**K1**	Hand washing is necessary before and after procedures are performed	Yes………………..………1<br>No ……………………..….2
**K2**	Can gloves provide complete protection against transmission of infections?	Yes………………..………1<br>No ……………………..….2
**K3**	All needles should be recapped after injection	Yes………………..………1<br>No ……………………..….2
**K4**	Use of an alcohol based antiseptic for hand hygiene is as effective as soap and water if hands are not visibly dirty	Yes………………..………1<br>No ……………………..….2
**K5**	Gloves should be worn if blood or body fluid exposure is anticipated	Yes………………..………1<br>No ……………………..….2
**K6**	Waste should not be segregated at the point of generation	Yes………………..………1<br>No ……………………..….2
**K7**	Tuberculosis (TB) is carried in airborne particles that are generated from patients with active pulmonary tuberculosis	Yes………………..………1<br>No ……………………..….2
**K8**	There is no need to change gloves between patients as long as there is no visible contamination	Yes………………..………1<br>No ……………………..….2
**K9**	Do you know how to prepare 0.5% chlorine solution?	Yes………………..………1<br>No ……………………..….2
**K10**	Safety box used should not be used when three quarters full	Yes………………..………1<br>No ……………………..….2

SECTION C – Practice of Infection Prevention Control

S/N	Question	Options
**P1**	Do you wash hands with soap before and after patient care?	Yes……………………..………1<br>No ……………………….….2
**P2**	I wash my hands with soap under running water for about 1 minutes	Yes……………………..………1<br>No ……………………….….2
**P3**	I wear a disposable face mask when attending to clients	Yes……………………..………1<br>No ……………………….….2
**P4**	I treat every patient as a potentially infectious client	Yes……………………..………1<br>No ……………………….….2
**P5**	I use alcohol based hand rub immediately after the removal of gloves	Yes……………………..………1<br>No ……………………….….2
**P6**	I recap needles before disposing them	Yes……………………..………1<br>No ……………………….….2
**P7**	I dispose of all potentially contaminated materials into an impermeable bag	Yes……………………..………1<br>No ……………………….….2
**P8**	I use Infection prevention guideline/evidence in my practice	Yes……………………..………1<br>No ……………………….….2

Thank you for participating!!!
