# Supplementary material for: Knowledge and determinants of infection prevention and control compliance among nurses in Yendi municipality, Ghana
Source: PLoS One. 2022 Jul 20;17(7):e0270508. doi: 10.1371/journal.pone.0270508 (PMC9299325; doi:10.1371/journal.pone.0270508)
Supplement: Attachment — Submitted filename: Response to review comments.docx [file pone.0270508.s004.docx]

**Response to Comments by Academic Editor**

| **Number** | **Comment** | **Action taken** |
| --- | --- | --- |
| 1. | The Abstracts’ methods section should specify the maximum scores calculated for knowledge, compliance, etc. This would help the readers in interpreting for example whether the 7.26 score mentioned in the Results is high or low. | Thank you for the direction. We have provided these values in Line 29 and 30 of the ‘Manuscript with highlighted changes’ file. |
| 2. | Line 32: Please mention in the text what the number after ± represents (i.e., SD) | The sentence has been rephrased to capture ‘standard deviation’ as well. i.e., line 34 |
| 3. | Line 35, 258 and table legends: Please define the OPD abbreviation. | In line 37, the sentence has been reconstructed to give full meaning to OPD and in table legends, as directed. |
| 4. | Lines 36, 40, and 350: Phrasing should be refined, since it currently sounds a bit odd. Of course, a guideline should exist in order for it to be followed. The full text manuscript clarifies on line 119 that this refers to whether or not specific IPC guidelines existed in the department. This clarification should also be added to the Abstract. | Thank you for pointing this out. We have recognized the relevance of making clarification to this and has incorporated that into lines 39, 40 and 44 of the Abstract section. |
| 5. | Line 66: Please revise the term “modest”. | Thank you for the comment. The word ‘modest’ has been replaced with ‘appropriate’ in line 69. |
| 6. | Overall, the manuscript’s introduction would benefit from more structuring, to focus only on HAIs. Right now, there is a mix, for example, line 75 mentions communicable diseases in general, and lists COVID-19 as example, without mentioning whether there is any connection between this phrase and the rate of COVID HAIs. | Thank you for pointing this out. The introduction section of the manuscript has been restructured to have a uniform focus on HAIs. |
| 7. | Line 86: Please revise this statement: “This study employed a descriptive quantitative cross-sectional study” – this appears to have been a cross-sectional survey. Please avoid repeating the term “study” and please revise the use of the term “quantitative”. | Thank you. In lines 91 and 92, we have carefully considered this and the whole sentence has been rephrased and the use of ‘quantitative’ revised. |
| 8. | What is the relevance of including lines 94-95 in the Methods section? They could at best be used to bring an argument as to why this study was needed in the Introduction section, or in the Discussion section, but they are not a good fit for the Methods section. | Thank you for pointing this out. We have considered this direction and the sentence has been rephrased to be a better fit in lines 81 and 82 of the Introduction section. |
| 9. | Line 107: Please define: Theatre. | In line 124, the word theatre has been defined as ‘surgical operating room’ |
| 10. | Line 174: Please correct: “date” to “data”. | Correction has been made in line 199 |
| 11. | Please start the Results section by specifying the rate of non-response in total and by department/level of qualification. | Thank you for the comment. This direction has been implemented (lines 202 to 204) |
| 12. | Line 207: Please correct “a thirds” to “one third”. | Correction made (line 226.) |
| 13. | Line 207-208: There probably is an error in phrasing here, and you were referring to the percentage of respondents who correctly reported that recapping should not be performed, as can be deduced from Table 2. | Thank you for pointing this out. We actually meant the negative form of the statement, however, typographically omitted the word ‘not’ in the sentence. This has been rephrased to reflect the true position of the authors. |
| 14. | Line 233: Please correct “rob” to “rub”. | Correction made; line 243 |
| 15. | Line 281: Phrasing is not very clear, please elaborate: “right attitudinal posture”. | Thank you for this comment. We have rephrased the statement to refer to attitudes towards compliance to IPC protocols. The sentence now reads “High compliance could also be plausibly due to the good attitudes of these highly knowledgeable nurses towards adherence to IPC protocols.” |
| 16. | Line 284: What is the connection with students? | Thank you for pointing this out. We actually meant to write ‘nurses’ and not students. Hence, students have been replaced with nurses in line 293. |
| 17. | Line 308: Please revise the term “harbors”. | Thank you for the comment. The word ‘harbors’ has been replaced with ‘includes’ in line 322. |
| 18. | Line 330: Please rephrase “pasted”. | The word ‘pasted’ has been replaced with ‘posted’(line 351) |
| 19. | Throughout the discussion, there is a repetition that further studies should be conducted, i.e., lines 296-297 and 321-322, and line 347. I would suggest that instead of comparing the results so much with field literature, you focus on highlighting on what your findings mean for your specific setting and how these could be generalized, or not, to a wider setting in your country. | Thank you for the comment. As suggested, we have elaborated on the practical implications of our findings to the study setting;  We have replaced lines 296-297 with “This finding can be explained that more males (54.7%) than females (45.3%) demonstrated high compliance to IPC protocols in this study. This probably contributed to the statistical difference observed between gender and compliance to IPC protocols in this study. Therefore, the findings of this study may not be generalisable to other populations.” Also, lines 321-322 have been replaced with “Diploma holding nurses greatly outnumber the degree holders in Ghana. Therefore, this disproportionate representation of the different cadre of nurses could have influenced the association between cadre and IPC compliance. Given the nationalistic picture of the proportions of diploma nurses relative to degree nurses, the findings of this study could be generalised to other nursing populations in Ghana.” For line 347, we have deleted the statement “We therefore recommend that future research should conduct a longitudinal study using larger sample sizes.” However, as rightly pointed out, the practical and research implications of our findings had already been explained. |
| 20. | Lines 339-341 would be a better fit in the Methods section. | Thank you for pointing this out. We have considered the statement and it has been moved to ‘Validity and Reliability’ section of the Methods, in line 182. |
| 21. | Line 353: Please check spelling for guidelines. | Spelling checked and correction made. (Line 372) |
| 22. | Lines 361-364: Please confirm that all persons mentioned here have consented to have their name published. | All individuals whose names are mentioned in the manuscript have granted consent for their names to be published. |
| 23. | Please check that all the information in the supplementary files provided is anonymized and cannot be considered as identifiable data. | The information in the supplementary files provided, especially survey and pre-test data has been cross-checked to ensure it does not include, either names, telephone or address of any participant.  Thank you for the comments. |

**Response to Comments by Reviewer #1**

| **Number** | **Comments** | **Action taken** |
| --- | --- | --- |
| 1. | Abstract – In the methods section (line 29, 30), the authors should consider adding that the multivariate logistic regression was used. | Direction has been taken into consideration and added in to line 30 of the abstract. |
| 2. | Introduction- line 49- consider inserting ‘associated with high’ before the expenditure if it explains better what the authors intend to communicate. | Recommendation added in to line 52 |
| 3. | It is not clear what the inclusion and exclusion criteria are. | We appreciate the comment. the inclusion and exclusion criteria have been provided. Line 114 to 118   - The inclusion criteria for this study considered professional nurses of all cadre, who were licenced and permanently employed in the facility. - However, student nurses on clinical attachment and all nurses on national service were exempted from participating in the study. |
| 4. | It is unclear whether Line 95 is referring to the hospital or the entire municipality. The authors should consider specifying where exactly the inadequacy/lack of resources is referring to. | Thank you for pointing this out. We actual meant health service within the municipality as a whole. This clarification has been indicted in line 83 to 85. |
| 5. | A summary of the numbers obtained for the different cadre of professionals (from the various units) after the selection based on the probability proportional to strata approach for selection would be helpful. | This recommendation has been considered. A Table has been used to summarize that. i.e., Table 1.  Also, further details on number of participants from various units/departments has been presented in Table 2. |
| 6. | Consider rephrasing line 133-134 – to ‘Frequencies and percentages were used to summarize data on the socio-demographic characteristics, knowledge and compliance to IPC guidelines. | Line 154 to 155 has been rephrased to omit descriptive statistics and include ‘frequencies and percentages’ |
| 7. | Line 140 – There is no need to include median (with IQR) since the authors ascertained that the data was normally distributed. | The Table has been removed and reports on overall knowledge and compliance to IPC, reported in prose only. |
| 8. | The authors would need to explain why statistical significance was set at p < 0.20 at the bivariate analysis stage. | Thank you for pointing this out. The decision to use threshold of a p-value < 0.20 is based on the discussion in literature on ‘Variable selection strategies and its importance in clinical prediction modelling.’ (Chowdhury and Turin, 2020). This has also been demonstrated by; Abukari et al. (2019) in their “Maternal determinants of low birth weight and neonatal asphyxia in the Upper West region of Ghana”.  Line 176 to 177 has been rephrased to incorporate these citations. |
| 9. | Consider rephrasing line 150 as it is currently confusing. The previous comment above may provide a guide. | Thank you for the comment. the sentence has been rephrased. |
| 10. | Line 163 – Cronbach is a name so should start with upper case. | Line 192 has been reviewed to start the name with capital C. |
| 11. | • Table 1  o The unit of age, i.e., years, should be included in the table  o The NMO should also be explained in the footnote | The unit of age has been provided in brackets close to age in Table 2.  All abbreviations including NMO has been defined in the footnote |
| 12. | Line 207 – Consider starting the sentence with ‘Less’. | Direction considered and line 228 reviewed. |
| 13. | Line 207- it is unclear the type of recapping the authors is describing as ‘aptly reported’. The authors should bear in mind that if the recapping is done holding the used needle and the cap in one’s hands, it would be very dangerous and an inappropriate IPC practice. This needs further clarification. | Thank you for pointing this out. The authors actually meant the negative form of the statement, however, typographically omitted the word ‘not’ in the sentence. This has been rephrased to reflect the true position of the authors. |
| 14. | Table 2 – The ‘No’ column is implied since those are the only two options. The authors should consider deleting the column and presenting the % separately as was done with Table 1. | Thank you for the comment.  The table presents results of binary categorical variables (Yes or No response). The responses obtained for each variable is independent of the responses for the other variables being assessed. Given that the responses do not present multiple choices, we think that it is not out of place to report both “Yes” and “No” which sum up to 100%. Furthermore, considering that some vital responses that were majorly represented as “No” would lose statistical significance, if not reported, we think that presenting both “Yes” and “No” is apt. |
| 15. | Line 231 – According to the WHO, the recommended duration of the entire handwashing procedure should be 40-60 seconds. In this study, the authors seem to have considered 1 minute (60 seconds) as the ideal. The researchers would need to provide justification (and reference) for this choice. | We intended to mean the same thing. However, upon your review and direction, we have rephrased the statement (line 240) to reflect the WHO’s position on that.  Thank you for the correction. |
| 16. | Table 3 – Similar comment as for Table 2. | Thank you for the comment. As explained earlier, the table presents results of binary categorical variables (Yes or No response). The responses obtained for each variable is independent of the responses for the other variables being assessed. Given that the responses do not present multiple choices, we think that it is not out of place to report both “Yes” and “No” which sum up to 100%. Furthermore, considering that some vital responses that were majorly represented as “No” would lose statistical significance, if not reported, we think that presenting both “Yes” and “No” is apt. |
| 17. | Consider describing the content of Table 4 in prose only and deleting Table 4. | Direction has been considered, Table 4 removed and reports on overall knowledge and compliance to IPC reported in prose only. |
| 18. | The authors should consider reconciling the terminologies used in the write up on the data analysis with that used in the write up on the overall knowledge level and compliance (lines 241-246) - good versus High level; poor versus low | Thank you for this correction. The authors have reviewed this and has considered using the words ‘high’ and ‘low’ consistently throughout the report. |
| 19. | Line 260-261 – The authors should consider explaining the different nursing training and qualifications so that the global readers can understand when ‘diploma or certificate’ is used. This can be explained in the study population section. | Thank you for the input. This information will be necessary to aid global readership’s understanding. As such, we have provided that in the report. Line 100 to 104 contains that. |
| 20. | • Line 270-271. The reasons given may or may not be the case and the authors should consider rephrasing to reflect this. | Thank you for the comment. We have rephrased the statement by adding "probably". The statement now reads “This implies that majority of these nurses probably received adequate insight regarding IPC, through the education and training curricular of their institution.” |
| 21. | • Line 276-279. The authors should consider rewording the statement so that the current study findings are the focus before any comparisons are carried out, as was done in lines 267-270. | We are grateful for the comment. We have refocused attention on the findings of the current study. The statement now reads “This finding is consistent with the report of Russell et al. [26] in North-eastern US, Mitchell et al. [27] in Australia and Ampadu [28] in Ghana” |
| 22. | Line 284- 285. Consider rephrasing to reflect the need for structures to promote the adherence to IPC protocols. | Thank you for the comment. We have rephrased the statement to read “Consequently, we recommend that management of healthcare facilities should put in place structures such as handwashing stations, that promote IPC and instil discipline towards adherence to such instituted IPC measures.” |
| 23. | • Line 293 – Consider inserting ‘of HAIs’ after transmission and ‘these’ before infections. | Thank you for the comment. We have inserted “of HAIs” and “these” as suggested. The statement now reads “Compliance to IPC protocols reduces the transmission of HAIs, which reduces morbidity, disability and mortalities, associated with these infections among healthcare workers as well as the patients they nurse.” |
| 24. | Lines 323. Consider using ‘department where the participant/nurses worked’ instead of ‘working department’. | Thank you for the comment. “Department where the participant/nurses worked” has now been used in place of “working department”. The statement now reads “Finally, the presence of IPC guides in department where the participant/nurses worked was associated with higher likelihood of complying with the provisions of the guidelines.” |
| 25. | o Lines 341 – 343. Consider rephrasing the sentence and deleting the phrase on what multistage sampling is. | Thank you for the comment. We have rephrased the statements to read “Our study used multistage stratified random sampling technique to sample the study participants. This therefore, improves the external validity and hence representativeness of the study [24].” |
| 26. | o Line 344 – the authors should provide information on what the power of this study is. | We are grateful for this comment. We have deleted the “power” aspect mentioned earlier because no power analysis was done in this study. The statement now reads “However, although our study administered a representative sampling technique, we cannot completely vouch for its representativeness [24].” |
| 27. | o Line 347- consider ending the sentence with ‘as well as more objective ways of gathering information on compliance to IPC protocols.’ | Thank you for this comment. The last sentence being referred to has been revised based on the editor’s comment. However, we agree with the reviewer’s suggestion as well. Therefore, in an attempt to satisfy both laudable comments, we have reviewed the statement which now reads “Therefore, future research should focus on more objective ways (such as a longitudinal design) of gathering information on compliance to IPC protocols.” |
| 28. | • Check and update references according to the journal’s guidelines. Some of the references are incomplete. | All intext citations have been duly listed in the bibliography, to contain all relevant features according to the journal’s requirements. |
| 29. | • Also check reference 10 and 16 | Full bibliographies have been provided. Kindly see reference number 10 and 15 in the bibliography. |

**Response to Comments by Reviewer #2**

| **Number** | **Comment** | **Action taken** |
| --- | --- | --- |
| 1. | In terms of sample size calculation, the authors used Yamane's formula, which assumes a prevalence of 50% as that of the main outcome. Authors should provide the unit of AGE (years) in the tables (line 199 and 264) | Thank you for the comment. The unit for age (years) has been provide in brackets next to age in both tables. |
